# Infectious diseases at the front door: Focus on the fundamentals

**DOI:** 10.1016/j.clinme.2024.100268

**Published:** 2024-11-27

**Authors:** Tim Crocker-Buque, Ponnusamy Saravanan

**Affiliations:** a The Peter Doherty Institute for Infection and Immunity, Melbourne, Victoria, Australia; b Warwick Applied Health, Warwick Medical School, University of Warwick, Coventry, United Kingdom; c Diabetes, Endocrinology and Metabolism, George Eliot Hospital NHS Trust, Nuneaton, United Kingdom

As we are drawing close to 2024, it is my pleasure to write a joint editorial with Dr Crocker-Buque for this year’s final issue of *Clinical Medicine*. While the COVID-19 pandemic drew the world’s attention towards one infectious disease, we must not lose focus on the ‘slower pandemics’ that have continued unabated in the background. We picked four articles from this issue to bring these challenges back to the forefront, emphasising the fundamentals of common infectious diseases that we encounter in acute medical practice worldwide.

Antimicrobial resistance, a global healthcare threat, continues to grow mostly unnoticed. Clinicians overprescribe antibiotics, often unnecessarily and for prolonged duration, due to habit and heavy workloads. O’Gorman *et al*^1^ highlight simple and effective tools for improving antimicrobial stewardship that should be a standard everywhere. The clinical cases by Hayton and Wickramasinghe^2^ complement this by demonstrating how judicious diagnostics can curb unnecessary antibiotic use.

*Staphylococcus aureus* remains a common and deadly pathogen. While the infectious disease community awaits with bated breath the results of the SNAP trial on *S aureus* bacteraemia treatment,^3^ patients still require rapid diagnosis, early investigation and effective antibiotics. Shah and Baltas’s article is a critical reminder of how *S aureus* can seed throughout the body and present with diverse clinical syndromes.^4^

Malaria, a bellwether for many African health systems, remains the most common imported tropical infection in the UK. Progress in reducing disease burden has stagnated over the past decade, with increases in incidence and mortality since COVID-19.^5^ As revolutionary malaria vaccines for children are rolled out, first malaria exposure, with higher risk of severe disease, may shift to older age groups. Salkeld *et al* remind us that a prompt blood film enables lifesaving early treatment,^6^ provided clinicians remember to request it.

While tackling antibiotic resistance demands a consistent and coherent long-term global approach, by adhering to simple principles and focusing on the fundamentals, we can reduce avoidable morbidity and mortality from common infections. This should be a priority for all healthcare professionals.

## Declaration of competing interest

We declare that we have no conflicts of interest.
